# Tanyu Tongzhi Formula Delays Atherosclerotic Plaque Progression by Promoting Alternative Macrophage Activation *via* PPARγ and AKT/ERK Signal Pathway in ApoE Knock-Out Mice

**DOI:** 10.3389/fphar.2021.734589

**Published:** 2021-12-13

**Authors:** Lan Ma, Xiaoce Dai, Chenxia Wu, Mingshuang Li, Hongzhuan Sheng, Wei Mao

**Affiliations:** ^1^ Department of Cardiology, Affiliated Hospital of Nantong University, Nantong, China; ^2^ The First School of Clinical Medicine of Zhejiang Chinese Medical University, Hangzhou, China; ^3^ Department of Cardiology, First Affiliated Hospital of Jiaxing University, Jiaxing, China; ^4^ Department of Cardiology, First Affiliated Hospital of Zhejiang Chinese Medical University, Hangzhou, China; ^5^ Key Laboratory of Integrative Chinese and Western Medicine for the Diagnosis and Treatment of Circulatory Diseases of Zhejiang Province, Hangzhou, China

**Keywords:** Tanyu Tongzhi formula, atherosclerosis, traditional Chinese medicine, alternative macrophage activation, PPARγ

## Abstract

We previously demonstrated that the Tanyu Tongzhi Formula (TTF) significantly alleviated the clinical symptoms of patients with coronary heart disease and lowered serum lipid and inflammatory factor levels in patients with coronary heart disease and atherosclerosis model rats. However, the mechanism underlying TTF remains unknown. In this study, we examined the effect of TTF on atherosclerotic plaques in ApoE^-/-^ mice and underlying mechanisms involved in macrophage polarization. Sixty male ApoE^-/-^ mice were randomly divided into four groups. Mice in the control group were fed a regular diet, whereas experimental mice were fed a high-fat diet and received either saline (HFD group) or TTF at concentrations of 0.60 (TTF-L group) or 2.25 g/ml (TTF-H group) by daily oral gavage for 16 weeks. In the TTF-L and TTF-H groups, the levels of serum cholesterol, triglyceride, interleukin (IL)-1β, IL-6, and tumor necrosis factor (TNF)-α were decreased, lipid content was significantly decreased, and percentage area of collagen/lipid increased in atherosclerotic plaque compared to in the HFD group. Moreover, we found TTF promoted the expression of alternative macrophage markers (Fizz1, Arg1, and Mrc) and suppressed the expression of M1 macrophage markers (TNF-α, IL-1β, and IL-6) by regulating peroxisome proliferator-activated receptor γ (PPARγ) expression and AKT/extracellular signal-regulated kinase (ERK) activation. We further investigated whether alternative macrophage was reduced when PPARγ was inhibited or the AKT/ERK signaling pathway was activated. TTF delayed atherosclerotic plaque progression by promoting alternative macrophage activation through increasing PPARγ expression and inhibiting AKT/ERK phosphorylation, providing a theoretical basis for its clinical application.

## Introduction

Globally, the disability and fatality rate of cardiovascular diseases, such as coronary heart disease (CHD) and stroke rank first among non-communicable diseases, leading to large medical and economic burdens on society and individuals. Atherosclerosis (AS), the critical pathological basis of these diseases, is a chronic inflammatory disease characterized by narrowing of the blood vessels. The occurrence and development of AS are characterized by accumulation of various immune cells, release of different inflammatory factors, and formation of lipid and necrotic cores. Continued cell influx and proliferation lead to the more advanced lesions (Ross, 1993). Macrophage aggregation in the endothelium is a key indication of AS (Vergallo and Crea, 2020). Stimulation of different inflammatory factors in the microenvironment of the lesion induces macrophage polarization. When the number of inflammatory M1 macrophages increases and anti-inflammatory alternative macrophages decrease, the lesion progresses. In contrast, the plaque tends to be stable (Ross et al., 2014; Tabas and Bornfeldt, 2016). Macrophage polarization and inflammation, lipid metabolism, cell proliferation, and differentiation-related signaling pathways have been widely examined in the context of AS (Murray, 2017; Lin et al., 2018).

The treatment of phlegm and blood stasis has a long history in the clinical practice of traditional Chinese medicine for CHD. A large number of clinical and basic research findings have shown that the treatment could effectively improve clinical symptoms, inhibit plaque progression, reduce inflammatory response and protect cardiovascular system in CHD patients and AS animal models (Xie et al., 2011; Zhe et al., 2013; Liu et al., 2014; Ren et al., 2014; Deng et al., 2021). Our research team has carried out clinical and basic research on the treatment of phlegm and blood stasis and the efficacy indicators mainly include angina pectoris, ECG changes, lipid levels, insulin resistance, inflammatory indicators (Hua et al., 2008; Liu et al., 2008a; Liu et al., 2008b; Miao et al., 2020a; Miao et al., 2020b). The Tanyu Tongzhi Formula (TTF) is a prescription based on the fundamental principle of treating phlegm and blood stasis simultaneously, produced in the first affiliated hospital of zhejiang chinese medical university and is derived from “Gualou xiebai Banxia Decotion” from the classic Chinese medicine “*Golden Chamber Yao lve*”; the latter formula has been adjusted for use in clinical practice for more than one thousand years (Zhang, 2016). TTF follows the compatibility principle of “monarch, minister, assistant and guide” and its effects at the cellular level have been demonstrated previously (Ma et al., 2019). The anti-inflammatory effect can be enhanced by combined administration with other drugs, reducing the cytotoxic effects of drugs. Previous studies confirmed the effectiveness of TTF on CHD in long-term clinical practice and relevant basic research (Liu et al., 2008b; Dai et al., 2010; Jiang et al., 2010; Cai et al., 2017; Cai H. et al., 2020). Additionally, TTF increased the protein expression of peroxisome proliferator-activated receptor γ (PPARγ), which participates in regulating alternative macrophage activation in the monocytes of patients with CHD (Liu et al., 2008b; Dai et al., 2010). In this study, we explored whether TTF can alleviate the progression of atherosclerotic plaques and its underlying mechanism in regulating macrophage polarization.

## Materials and Methods

### TTF Preparation

The aqueous extract of TTF was provided by Huisong Co., Ltd. (Hangzhou, China). TTF contains 15 g *Trichosanthes* kirilowii Maxim (Cucurbitaceae; Trichosanthis Fructus), 15 g Salvia miltiorrhiza Bunge (Lamiaceae; Salviae miltiorrhizae radix et rhizoma), 10 g Allium macrostemon Bunge (Amaryllidaceae; Bulbus Allii Macrostemonis), 5 g Leech (Hirudinidae; Hirudo), 10 g Acorus gramineus Aiton (Araceae; Acori Tatarinowii Rhizoma), 15 g Curcuma aromatica Salisb (Zingiberaceae; Curcumae Radix), 20 g Poria cocos [Polyporaceae; Wolfiporia cocos (F.A. Wolf) Ryvarden and Gilb], and 10 g Citrus × aurantium L (Rutaceae; Citri Reticulatae Pericarpium) (Table 1). All drugs in TTF were authenticated by Director Jinxia Wang and Minxia Zheng of the First Affiliated Hospital of Zhejiang Chinese Medical University (Hangzhou, China). Voucher specimens were deposited at the Herbarium of Zhejiang University. The procedure used was as follows: After soaking all drugs (100 g) in 1- L distilled water 2 times for 30 min, the mixture was boiled for 1 h. After collecting the filtrate, an additional 1- L distilled water was added and boiled for 1 h, and the filtrate obtained twice was mixed and concentrated to 0.6 mg/ml and 2.25 g/ml, respectively. Hence, crude extracts at low and high concentrations were obtained.

**TABLE 1:** Components of TTF.

Chinese name	Place of origin	Family/Voucher number	Authority	Amount (g)
Quan Gua Lou	Shandong, China	Cucurbitaceae/Y.-J.Jin 1	Pharmacopoeia of China (2015)	15
Dan Shen	Anhui, China	Lamiaceae/Y.-J.Jin 2	Pharmacopoeia of China (2015)	15
Xie Bai	Shandong, China	Amaryllidaceae/Y.-J.Jin 3	Pharmacopoeia of China (2015)	10
Shui Zhi	Shandong, China	Hirudinidae/Y.-J.Jin 4	Pharmacopoeia of China (2010)	5
Shi Chang Pu	Shandong, China	Araceae/Y.-J.Jin 5	Pharmacopoeia of China (2005)	10
Yu Jin	Zhejiang, China	Zingiberaceae/Y.-J.Jin 6	Pharmacopoeia of China (2015)	15
Fu Ling	Zhejiang, China	Polyporaceae/Y.-J.Jin 7	Pharmacopoeia of China (2015)	20
Chen Pi	Guangdong, China	Rutaceae/Y.-J.Jin 8	Medicinal Plants in China (WHO, 1997)	10

### High-Performance Liquid Chromatography

The aqueous extract of TTF (200 µL) was added to 1 ml of 80% methanol, swirled, and shaken for 1 min. High-speed centrifugation was performed at 14,000 rpm for 10 min, and the supernatant was collected. The supernatant was filtered through a 0.22-µm filter membrane. Chromatographic analysis collection and integration of uracil, hypoxanthine, adenosine, lithospermic acid B, lithospermic acid, tanshinone I, and quercetin in TTF were evaluated using Xcilabur4.0 software (Thermo Fisher Scientific, Waltham, MA, United States). A QExactive high-resolution mass spectrometer (Thermo Fisher Scientiic) coupled with an UltiMate 3000 RS (Thermo Fisher Scientific) was employed for chemical identification.

Mass spectrometry analysis was performed in both positive and negative modes under the following parameters: scan range, 50.0–500.0 m/z; ion spray pressure, 3.2 kV (positive); capillary temperature, 300°C. The chromatographic conditions were as follows: chromatographic column, RP-C18 150 × 2.1 mm, Welch; flow rate, 0.300 ml/min; aqueous phase, 0.1% formic acid in aqueous solution (mobile phase A); organic phase, acetonitrile (mobile phase B); needle liquid, methanol; column temperature: 35°C; automatic sampler temperature, 10.0°C.; chromatographic gradient: 0–5 min, 2 %B; 5–10 min, 20 %B; 10–15 min, 50 %B; 15–20 min, 80 %B; 20–25 min, 95 %B; 25–30 min, 2% B.

### Animals

The study was approved by the Animal Ethics Committee of Zhejiang Chinese Medical University. Sixty male ApoE^-/-^ mice (6–8 weeks old, 18–22 g) in the C57/BL6 background were obtained from the Department of Laboratory Animal Science at Nanjing University. All mice were kept in a temperature-controlled room on a 12-h light/dark cycle with food and water available *ad libitum*. Animal experiments were carried out in accordance with the National Institutes of Health Guide for the Care and Use of Laboratory Animals (NIH Publications No. 8023, revised 1978).

All mice were adaptively fed for 1 week, after which high-fat diet feeding and TTF intragastric administration were started simultaneously. The mice were randomly divided into four experimental groups (15 mice per group): the control group was fed a common diet and HFD group was fed a high-fat diet (Research Diets, Inc, New Brunswick, NJ, United States; Lot: D12108C). The formulation of the high-fat diet is shown in Supplementary Table S1; the TTF-L and TTF-H groups were fed the HFD together with 0.2 ml low and high concentrations of TTF daily by oral gavage, respectively. After 16 weeks, the mice (n = 8–10 mice per group) were anesthetized with 1% sodium pentobarbital by intraperitoneal injection, and the hearts, aorta, and blood were harvested. The mice were perfused with normal saline through the heart before harvesting the tissues. Finally, the heart with the entire aorta and other tissues was isolated and stored at −80°C for subsequent histological evaluation or gene expression analysis.

### Serum Biochemical Analysis

Mice were fasted overnight, and then the retro-orbital sinus blood was collected, allowed to coagulate, and centrifuged at 1,000 ×*g* (RCF) at room temperature to isolate the serum. The serum was immediately frozen and stored at −80°C. The serum lipid profile, including cholesterol (CHOL), triglycerides (TG), low-density lipoprotein-cholesterol (LDL-C), and high-density lipoprotein-cholesterol (HDL-C), was measured at the Zhejiang Chinese Medical University with an AU 400 fully automated chemistry analyzer (Olympus, Tokyo, Japan) using the enzymatic-colorimetric method. Serum tumor necrosis factor (TNF)-α, interleukin (IL)-6, and IL-1β levels were evaluated by enzyme-linked immunosorbent assay according to the manufacturer’s instructions.

### Histological Evaluation of Atherosclerotic Lesions

The en face pinned aortas were stained with Oil-Red-O. Images of the aortas were captured using a stereoscope (Nikon, Tokyo, Japan). The proximal aortas attached to the heart were embedded in OCT compound and frozen at −80°C. The aortic root was serially sectioned into 7-μm sections from the site where the aortic valve appeared. A set of three consecutive sections was stained with Oil-Red-O and Masson’s trichrome for morphological analysis of atherosclerotic plaques. Macrophages and the expression of Arg1 in the aortic root were detected by immunofluorescence staining of sequential sections with rat anti–monocyte macrophage (CD68) monoclonal antibody (1 μg/ml, abcam, ab237968) and rabbit polyclonal anti-Arg1 (1:1,000, CST, 93668S) at 4°C overnight, followed by the corresponding secondary antibodies conjugated with Alexa Fluor® 488 and 647 (1:5,000, abcam, ab201844 and 1:5,000, CST, 43279S) for fluorescence detection. Images were captured using a Nikon A1R digital camera (Nikon). The sections were quantitatively analyzed using Image-Pro Plus 6.0 software (Media Cybernetics, Rockville, MD, United States).

### Cell Culture

Primary peritoneal macrophages (PMs) were isolated from successful modeling ApoE^-/-^ mice in the control, HFD, TTF-L, and TTF-H groups. Briefly, the mice were

Sacrificed by cervical dislocation, 4 ml of ice-cold RPMI medium was injected into the peritoneal cavity, the peritoneum was gently massaged for 1 min, the RPMI medium was collected and centrifuged at 800 ×*g* (RCF) for 5 min, and the PMs settled at the bottom of the test tube. The PMs were resuspended and cultured at 5% CO_2_ and 37°C for 2 h in RPMI containing 10% fetal bovine serum, nonadherent cells were removed, and the cells were cultured for subsequent assays.

### CCK-8 Assay

Ten microliters RPMI were added to each well of a 96-well plate, and then 90 μL PMs were seeded into the plates at a density of 5 × 10^4^ cells/well. After culturing for 2 h, nonadherent cells were removed and 90 μL RPMI containing TTF was added at gradient of concentrations for 24 h. Next, 10 μL CCK-8 reagent was added. The cells were placed in an incubator in the dark for 2 h. The optical density of the medium was measured using a microplate reader at a wavelength of 450 nm.

### Quantitative Real-Time Polymerase Chain Reaction

PMs isolated from ApoE^-/-^ mice were incubated with 0.5 mg/ml TTF for 6 h at 37°C and then incubated with 80 μg/ml oxidized LDL (ox-LDL; Yiyuan Biotechnologies, Guangzhou, China) for 24 h at 37°C. Total RNA was isolated from PM cells using TRIzol reagent (Invitrogen, Carlsbad, CA, United States). Total RNA (2 μg) was reverse-transcribed to cDNA using M-MLV transcriptase (Thermo Fisher Scientific) according to the manufacturer’s instructions. Real-time polymerase chain reaction was performed on an ABI 7500 System cycler (Applied Biosystems, Foster City, CA, United States) using SYBR Green PCR Master Mix (Transgen Biotic). The expression of six genes (Fizz1, Arg1, Mrc, TNF-α, IL-1β, and IL-6) was normalized against the expression of 18S rRNA. Data were analyzed using the 2^-ΔCt^ method. The primers used are shown in Table 2.

**TABLE 2:** **|** List of primers for quantitative Real-Time PCR.

Gene		Oligonucleotide sequence
Fizz1	Forward	TAC TTG CAA CTG CCTGTG CTT ACT
	Reverse	TAT CAA AGC TGG GTT CTC CACCTC
Arg1	Forward	CTCCAA GCC AAA GTC CTT AGA G
	Reverse	AGG AGC TGTCAT TAG GGA CAT C
Mrc	Forward	CAT GAG GCT TCT CCT GCT TCT
	Reverse	TTGCCG TCT GAA CTG AGA TGG
TNF-α	Forward	CCC​TCA​CAC​TCA​GAT​CAT​CTT​CT
	Reverse	GCT​ACG​ACG​TGG​GCT​ACA​G
IL-1β	Forward	GCA​ACT​GTT​CCT​GAA​CTC​AAC​T
	Reverse	ATC​TTT​TGG​GGT​CCG​TCA​ACT
IL-6	Forward	TTA​GTC​CTT​CCT​ACC​CCA​ATT​TCC
	Reverse	TTG​GTC​CTT​AGC​CAC​TCC​TTC
18S rRNA	Forward	GCA​ATT​ATT​CCC​CAT​GAA​CG
	Reverse	GGG​ACT​TAA​TCA​ACG​CAA​GC

### RNA-Sequencing and Gene Expression Analysis

PMs isolated from ApoE^-/-^ mice were incubated with 0.5 mg/ml TTF for 6 h at 37°C and then incubated with 80 μg/ml oxidized LDL (ox-LDL; Yiyuan Biotechnologies, Guangzhou, China) for 24 h at 37°C. The RNA-seq library was prepared for sequencing using standard Illumina protocols. Total RNA samples from ApoE^-/-^ PMs were isolated using TRIzol reagent. Library construction and sequencing were performed using BGI (Shenzhen, China). For data analysis, base calling was performed using SOAPnuke (v1.5.2). Clean reads were aligned to the genome using HISAT2 v2.0.5. Differential expression was determined using RSEM (v1.2.8), and the significance of differential gene expression was defined by DEGseq according to the combination of the absolute value of log2-fold-change ≥ 2 and *p* value ≤0.001. Gene Ontology and pathway annotation and enrichment analyses were based on the GO database (http://www.geneontology.org/) and Kyoto Encyclopedia of Genes and Genomes pathway database (http://www.genome.jp/kegg/), respectively. The R package heatmap was used for hierarchical cluster analysis of gene expression patterns.

### Western Blotting

Equal amounts of cells were subjected to sodium dodecyl sulfate-polyacrylamide gel electrophoresis, and western blotting analysis was performed using the mouse anti–CD68 antibody (1:1,000, abcam, ab955), rabbit anti-Arg1 antibody (1:1,000, CST, 93668S), rabbit anti-TNF-α antibody (1:1,000, CST, 3707S), mouse anti-PPARγ antibody (1:1,000, CST, 95128S), rabbit anti-extracellular signal-regulated kinase (ERK) antibody (1:2000, CST, 4370S), rabbit anti-phosphorylated (p)-ERK antibody (1:1,000, CST, 4695S), rabbit anti-AKT antibody (1:1,000,CST, 4685S), rabbit anti-p-AKT antibody (1:1,000, CST,4060S), and rabbit anti-β-actin antibody (1:2000, diagbio, db10001). Quantification of the band intensity was performed using ImageJ software 1.49v (NIH, Bethesda, MD, United States), and the values were normalized to the levels of β-actin.

### Statistical Analysis

Statistical analyses were performed using SPSS version 27.0 software (SPSS, Inc, Chicago, IL, United States). All results are presented as the mean ± standard error of the mean. Statistical significance was determined by *t*-test or one-way analysis of variance for data with a normal distribution and by Kruskal–Wallis test for non-normally distributed data or small samples. Statistical significance was set at *p* < 0.05. All cell experiments were performed at least 3 times.

## Results

### Quality Control of TTF

**FIGURE 1:**
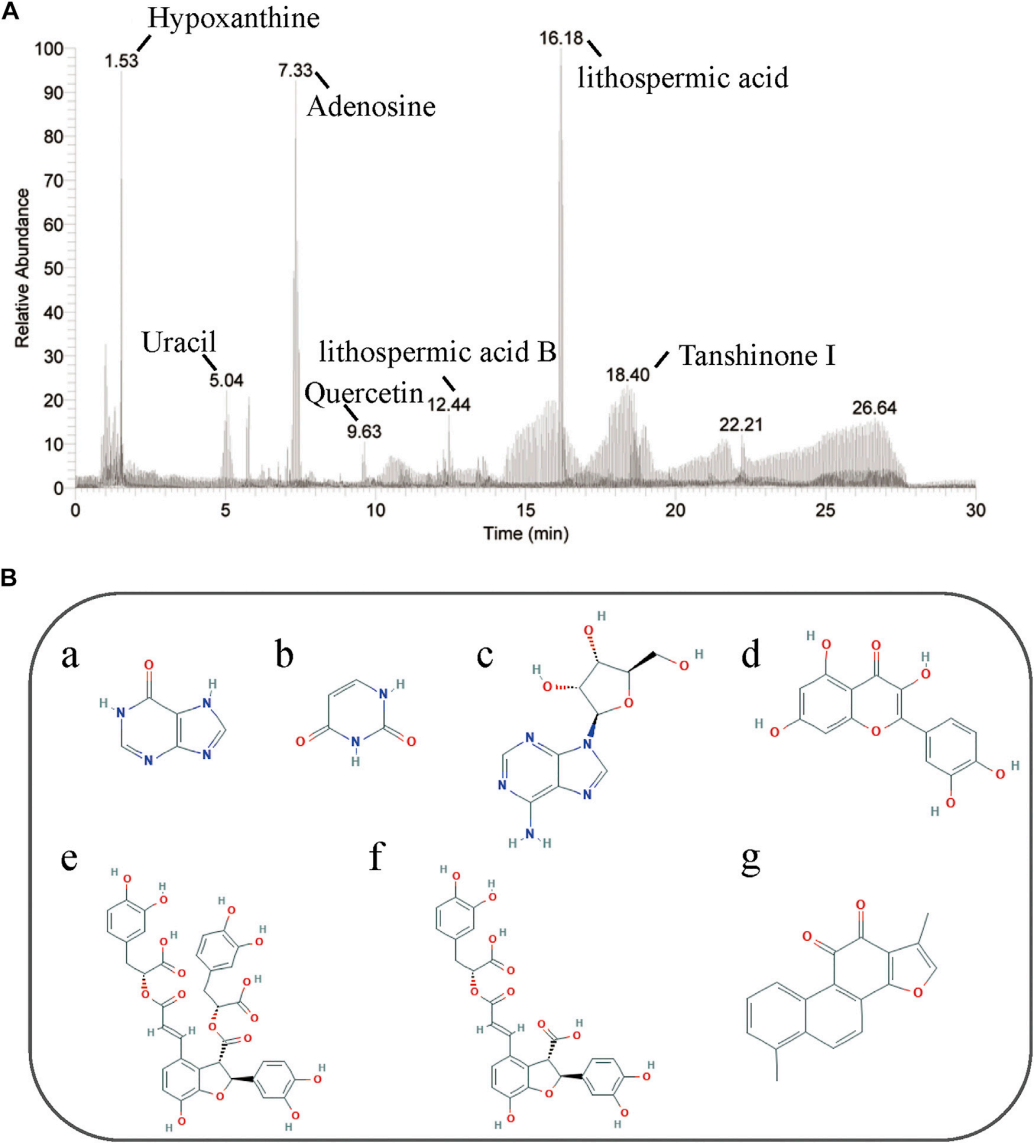
HPLC of TTF. **(A)** Seven chemical compositions in TTF were identified by HPLC. **(B)** Chemical structure of (a) hypoxanthine, (b) uracil, (c) adenosine, (d) quercetin, (e) lithospermic acid B, (f) lithospermic acid, (g) tanshinone I.

Seven chemical compositions were identified in TTF using high-performance liquid chromatography: hypoxanthine (observed m/z: 110.035, retention time (RT): 1.51), uracil (observed m/z: 113.034, RT: 1.57); adenosine (observed m/z: 268.104, RT: 7.33); quercetin (observed m/z: 153.018, RT: 10.13), lithospermic acid B (observed m/z: 719.160, RT: 13.59), lithospermic acid (observed m/z: 539.119, RT: 16.68); and tanshinone I (observed m/z: 279.099, RT: 17.73) (Figure 1). Our results showed that the quality of TTF met the pharmacopeia requirements, which provided a reliable and controllable sample for subsequent experiments.

### Effects of TTF on Plasma Lipid Profiles and Inflammatory Factors in ApoE^-/-^ Mice

**FIGURE 2:**
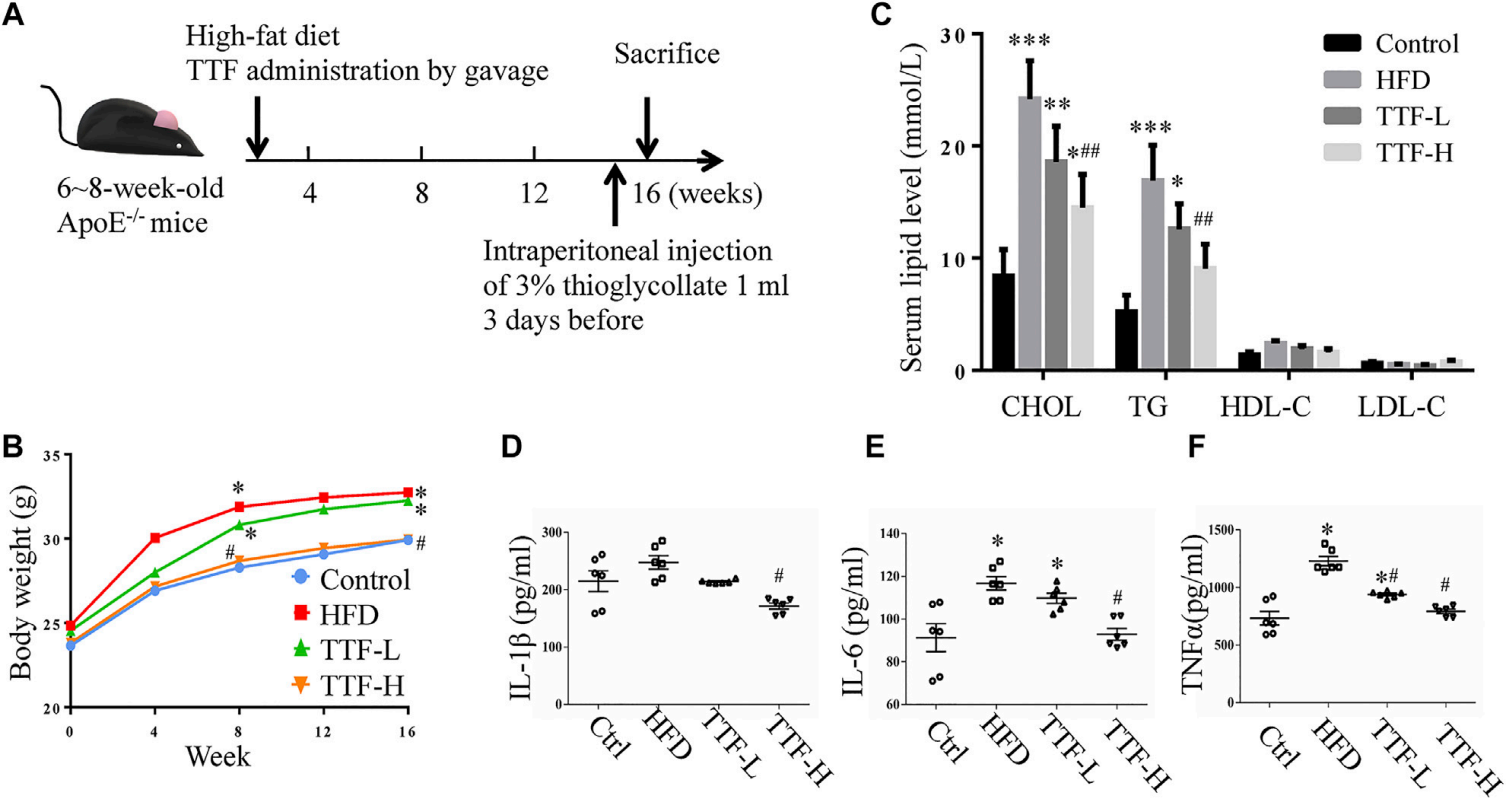
TTF affects body weight and serum lipid level in ApoE^-/-^ mice **(A)** Experimental protocol of the *in vivo* study **(B)** Curve of body weight of ApoE^-/-^ mice. *p < 0.05 vs Control group **(C)** Histogram of serum content of cholesterol, triglyceride, high density lipoprotein and low-density lipoprotein of ApoE^-/-^ mice **(D)** Histogram of serum content of IL-1β **(E)** IL-6, and **(F)**TNF-α in ApoE^-/-^ mice. Values are presented as means ± SEM (n = 10 per group). *p < 0.05 vs Control group, **p < 0.01 vs Control group, ***p < 0.001 vs Control group, ##*p* < 0.01 vs HFD group.

To investigate the role of TTF in atherosclerotic development, we established an AS model with ApoE^-/-^ mice fed a HFD for 16 weeks. Our experimental animal scheme is shown in Figure 2A. The body weight of mice in the TTF-H group was decreased compared with the control group and HFD group from the 8th week of modeling (Figure 2B). The serum lipid levels of mice in the four groups were measured; CHOL and TG levels in the HFD group were significantly higher than those in the control group (*p* < 0.001, *p* < 0.01). In the TTF-L group, the CHOL and TG levels piwere decreased compared to that in the HFD group, but the difference was not significant. However, the CHOL and TG levels in the TTF-H group were significantly lower than those in the HFD group (*p* < 0.01) (Figure 2C). The four groups showed no significant difference in LDL-C and HDL-C levels (all *p* > 0.1). Serum IL-6 and TNF-α levels were significantly higher in the HFD group than in the control group (*p* < 0.01). The levels of IL-1β, IL-6, and TNF-α in the TTF-H group were much lower than those in the HFD group (*p* < 0.01) (Figures 2D–F).

### Effects of TTF on Atherosclerotic Plaque Composition in ApoE^-/-^ Mice

**FIGURE 3:**
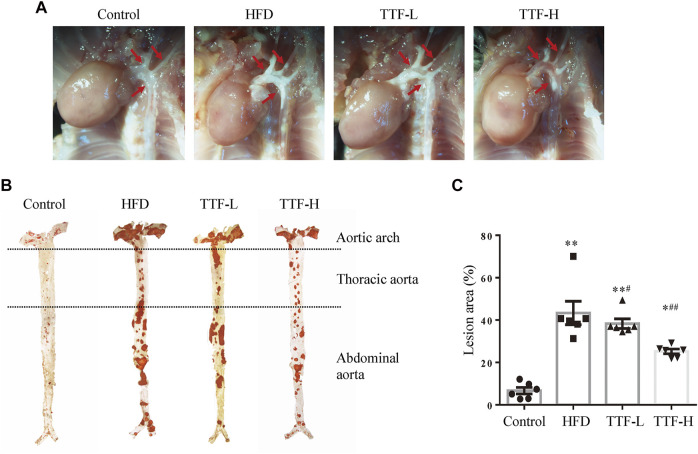
TTF relieves atherosclerotic plaque in ApoE^-/-^ mice **(A)** Representative *in vivo* images of aortic arch of ApoE^-/-^ mice. The arrows point the location of the AS plaque **(B)** The oil-red-O staining of en face aorta and **(C)** the quantitative analysis of atherosclerotic lesion area of whole aorta. Values are presented as means ± SEM (n = 6 per group). *p < 0.05 vs Control group, **p < 0.01 vs Control group, #*p* < 0.05 vs HFD group, ##*p* < 0.01 vs HFD group.

**FIGURE 4:**
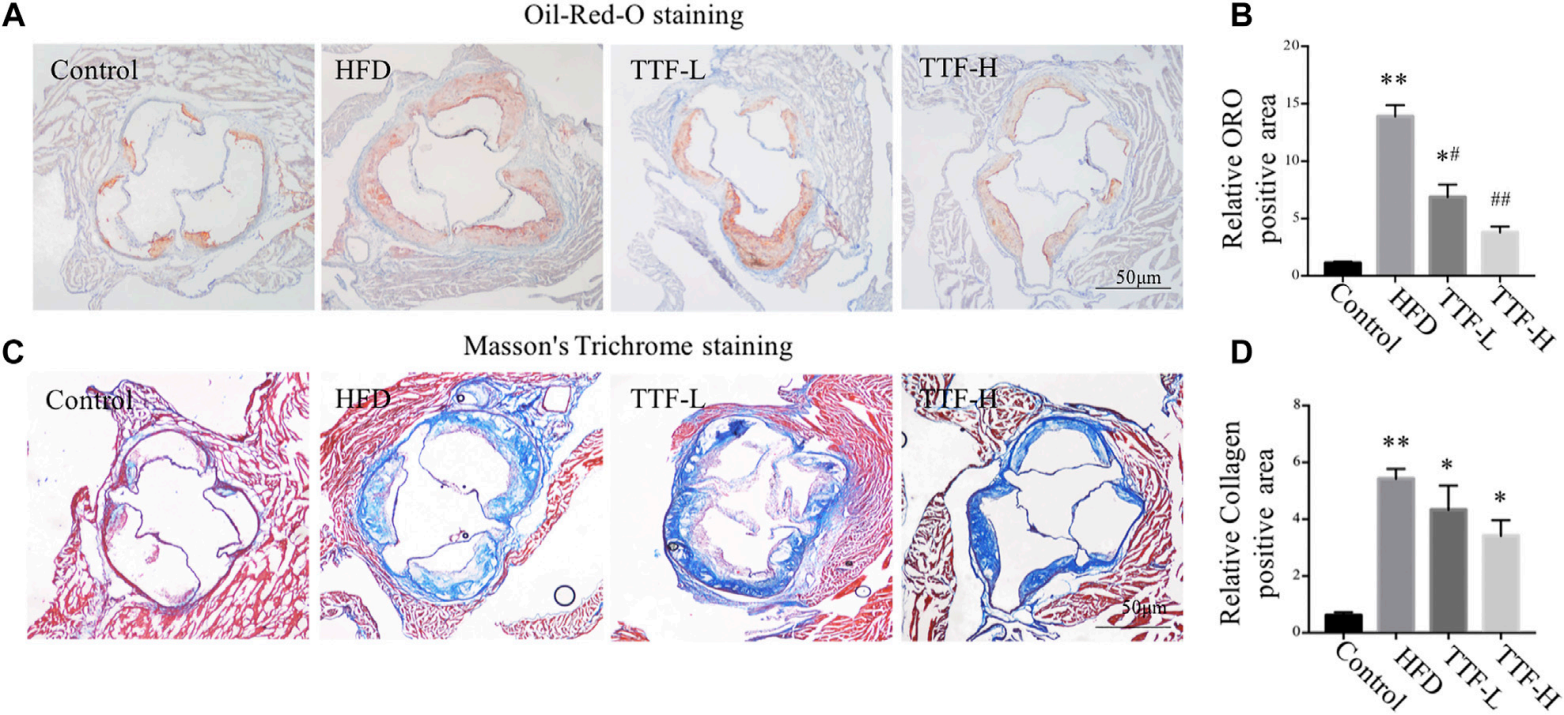
TTF reduces lipid content and increases collagen content in atherosclerotic plaque of ApoE^-/-^ mice. Representative images of **(A)** Oil-Red-O staining and **(B)** the quantitative analysis of relative ORO positive area. Representative images of **(C)** Masson’s Trichrome staining of aortic sinus and **(D)** the quantitative analysis of relative collagen positive area. Values are presented as means ± SEM (n = 3 per group). *p < 0.05 vs Control group, **p < 0.01 vs Control group, #*p* < 0.05 vs HFD group, ##*p* < 0.01 vs HFD group. Bar = 50 μm.

We assessed the plaque area and stability using Oil Red O and Masson’s trichrome staining. The vivisection of the aortic arch, brachiocephalic trunk, left common carotid artery, and left subclavian artery showed that the HFD significantly increased plaque area in ApoE^-/-^ mice, whereas TTF significantly decreased the plaque area in these mice (Figure 3A). Oil Red O staining of the en face aorta showed that the plaque area in the HFD group was significantly higher than that in the control group (*p* < 0.01). The plaque areas in the TTF-L and TTF-H groups were also higher than those in the control group (*p* < 0.01, *p* < 0.05); however, compared with the HFD group, they were significantly decreased (*p* < 0.05, *p* < 0.01) (Figures 3B,C). The results of Oil Red O staining of the aortic valve were consistent with those of the en face (Figures 4A,B). Masson’s trichrome staining was used to detect collagen deposition in the plaque region of the aortic valve. The collagen positive areas in the HFD, TTF-L, and TTF-H groups were significantly higher than that in the control group (*p* < 0.01, *p* < 0.05, *p* < 0.05), but they were slightly decreased in the TTF-L and TTF-H groups compared with the HFD group with no significant difference (Figures 4C,D). The staining results suggest that TTF can significantly reduce the lipids and slightly reduce the collagen content in atherosclerotic plaque, which is helpful to delay the progression of plaque (Rekhter, 1999; Baardman, et al., 2020).

### Effects of TTF on Regulating Alternative Macrophage Activation in the Atherosclerotic Plaque

**FIGURE 5:**
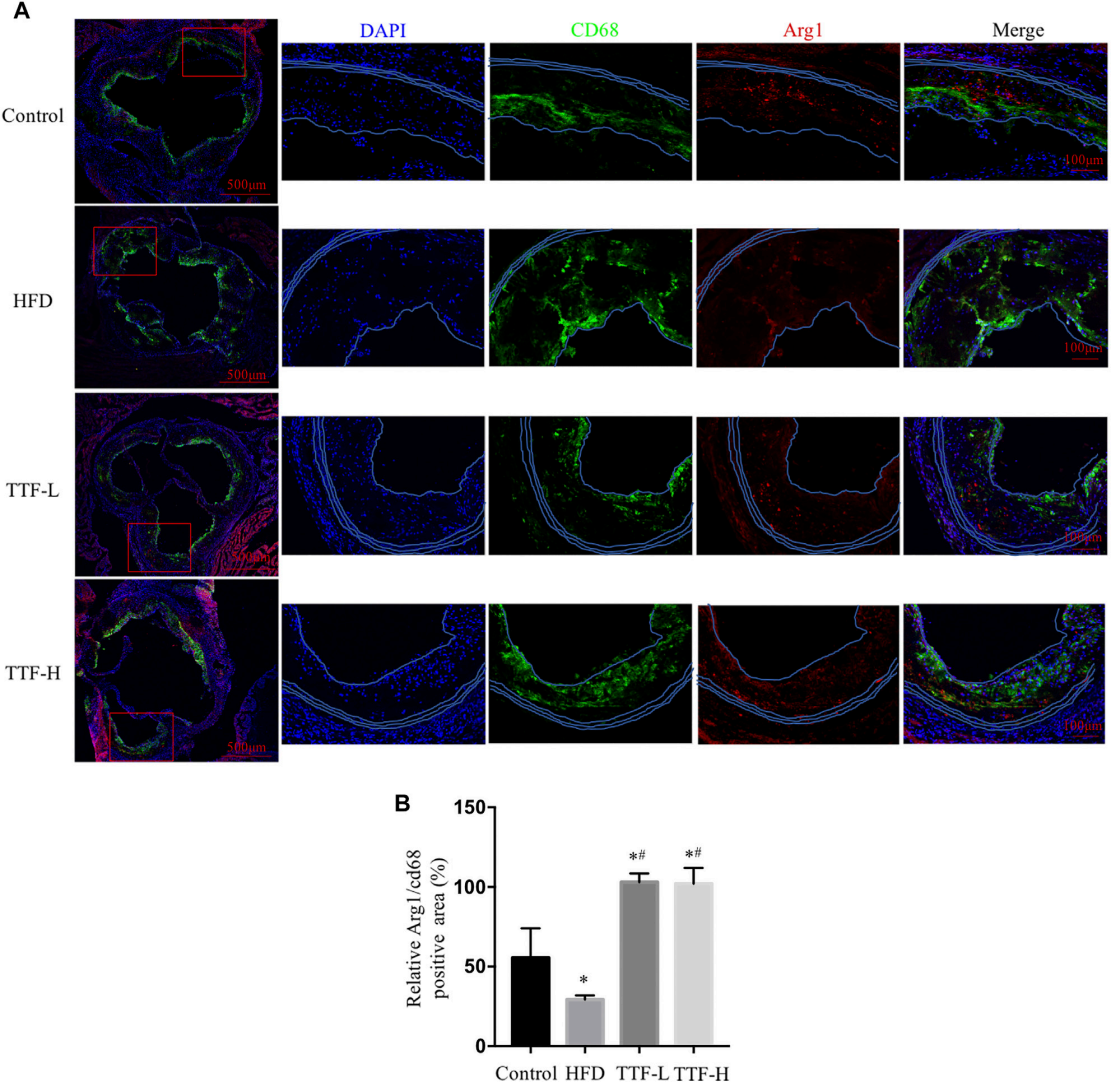
TTF increases the expression of alternative macrophage in atherosclerotic plaque of ApoE^-/-^ mice **(A)** Representative images of immunofluorescence staining of aortic sinus. Bar = 50 μm. Blue for DAPI, green for CD68 and red for Arg1 **(B)** the quantitative analysis of relative Arg1/CD68 positive area. Values are presented as means ± SEM (n = 3 per group). *p < 0.05 vs Control group, #*p* < 0.05 vs HFD group.

**FIGURE 6:**
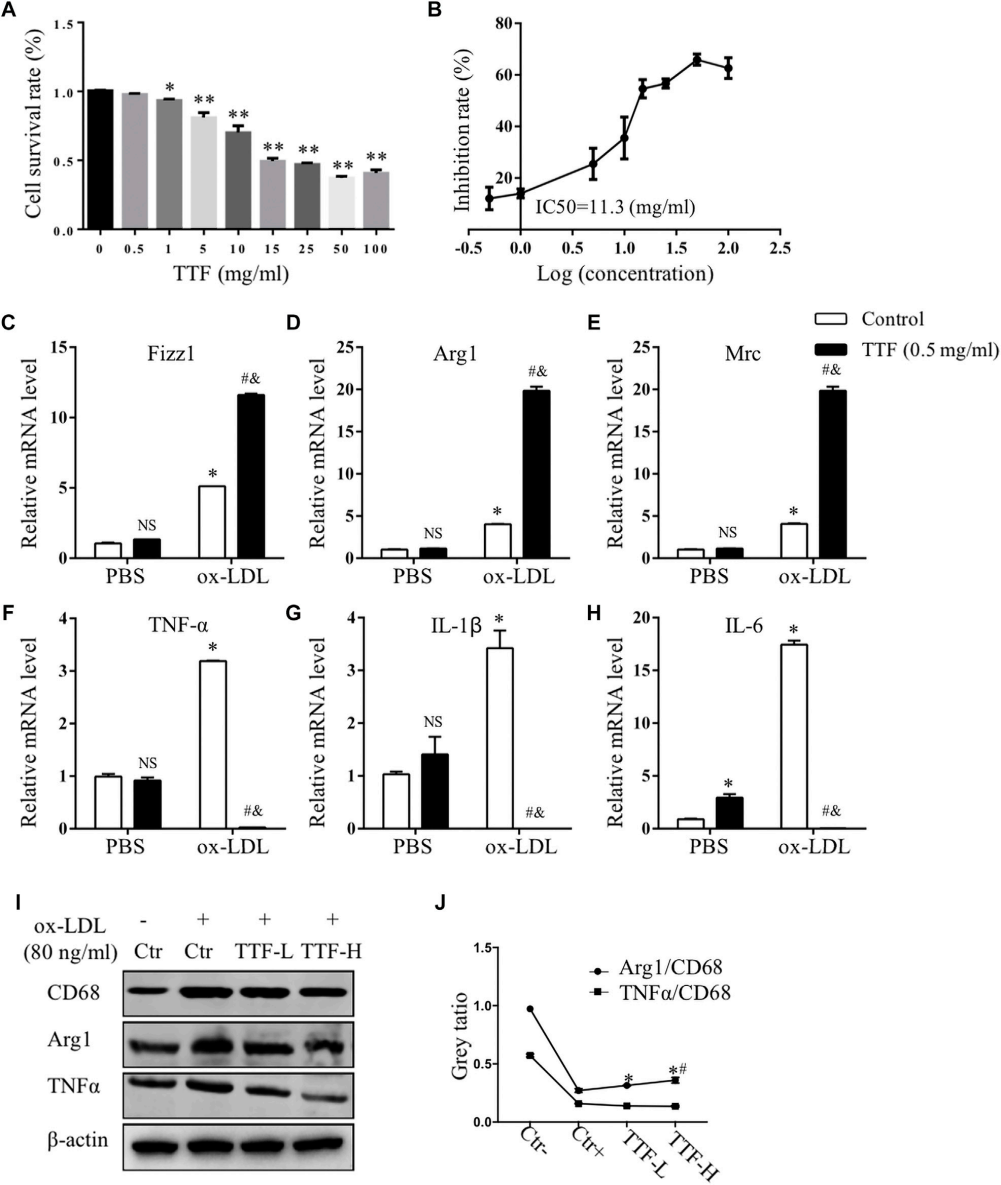
TTF affected the expression of mRNA of M1 and alternative macrophage markers. PM cells isolated from ApoE^-/-^ mice were treated with 0.5 mg/ml TTF for 24 h followed by ox-LDL stimulating for 24 h **(A)** The cell survival rate of PM pretreated with TTF and IC50 of PM **(B)**. *p < 0.05 and **p < 0.01 vs Control (TTF = 0 mg/ml) group **(C–E)** The mRNA expression of Fizz1, Arg1 and Mrc of alternative macrophage markers **(F–H)** The mRNA expression of TNFα, IL-1β and IL-6 of M1 markers. The relative mRNA level was measured by RT-qPCR analysis. Values are presented as means ± SEM (n = 3 per group). NS represents no significant with Control (PBS) group, *p < 0.05 vs Control (PBS) group, #*p* < 0.05 vs TTF (PBS) group, &*p* < 0.05 vs TTF (PBS) group **(I)** Western blotting showed the protein expression of CD68, Arg1, and IL-6 in PM cells treated with PBS, TTF-L (0.25 mg/ml), and TTF-L (0.5 mg/ml) stimulated with ox-LDL (80 μg/ml) **(J)** The quantitative analysis of grey ratio of Arg1/CD68 and TNFα/CD68. Values are presented as means ± SEM (n = 3 per group). *p < 0.05 vs Ctr + group, #*p* < 0.05 vs TTF-L group.

To investigate whether TTF regulated alternative macrophage activation in the atherosclerotic plaques of ApoE^-/-^ mice fed a HFD, we conducted immunofluorescence staining and analysis of Arg1 on aortic valve sections. Expression of the total macrophage marker CD68 in the HFD group was significantly higher, whereas expression of the alternative macrophage marker Arg1 was lower than that in the control group. In addition, compared with the HFD group, expression of CD68 in the TTF-L and TTF-H groups significantly decreased, and expression of Arg1 was increased (Figure 5A). The quantitative analysis of relative Arg1/CD68 positive area showed that the proportion of Arg1 positive macrophages in HFD group was significantly less than that in control group, while the proportion of arg1 positive macrophages in TTF-L and TTF-H groups were significantly higher than that in HFD group (Figure 5B). Moreover, to detect the expression levels of Macrophage markers in the peritoneal macrophages of ApoE^-/-^ mice by quantitative PCR assay, we determined the optimal drug concentration using the CCK-8 assay. The results showed that TTF (0.5 mg/ml) was an appropriate dosage for PMs (Figures 6A,B). Furthermore, quantitative PCR showed that TTF promoted mRNA expression of the alternative macrophage markers Fizz1, Arg1, and Mrc and decreased expression of the M1 macrophage markers TNF-α, IL-1β, and IL-6 following stimulation with ox-LDL (Figures 6C–H). Moreover, western blotting revealed significant up-regulation of the alternative macrophage-associated gene (Arg1) and down-regulation of the M1 macrophage-associated gene (TNF-α) in PMs treated with 0.25 and 0.5 mg/ml TTF (Figure 6I). Analysis of the grayscale image showed that Arg1/CD68 levels were significantly higher than TNF-α/CD68 levels in the TTF-L and TTF-H groups (Figure 6J).

### Effects of TTF on RNA Expression of Macrophages in ApoE^-/-^ Mice

**FIGURE 7:**
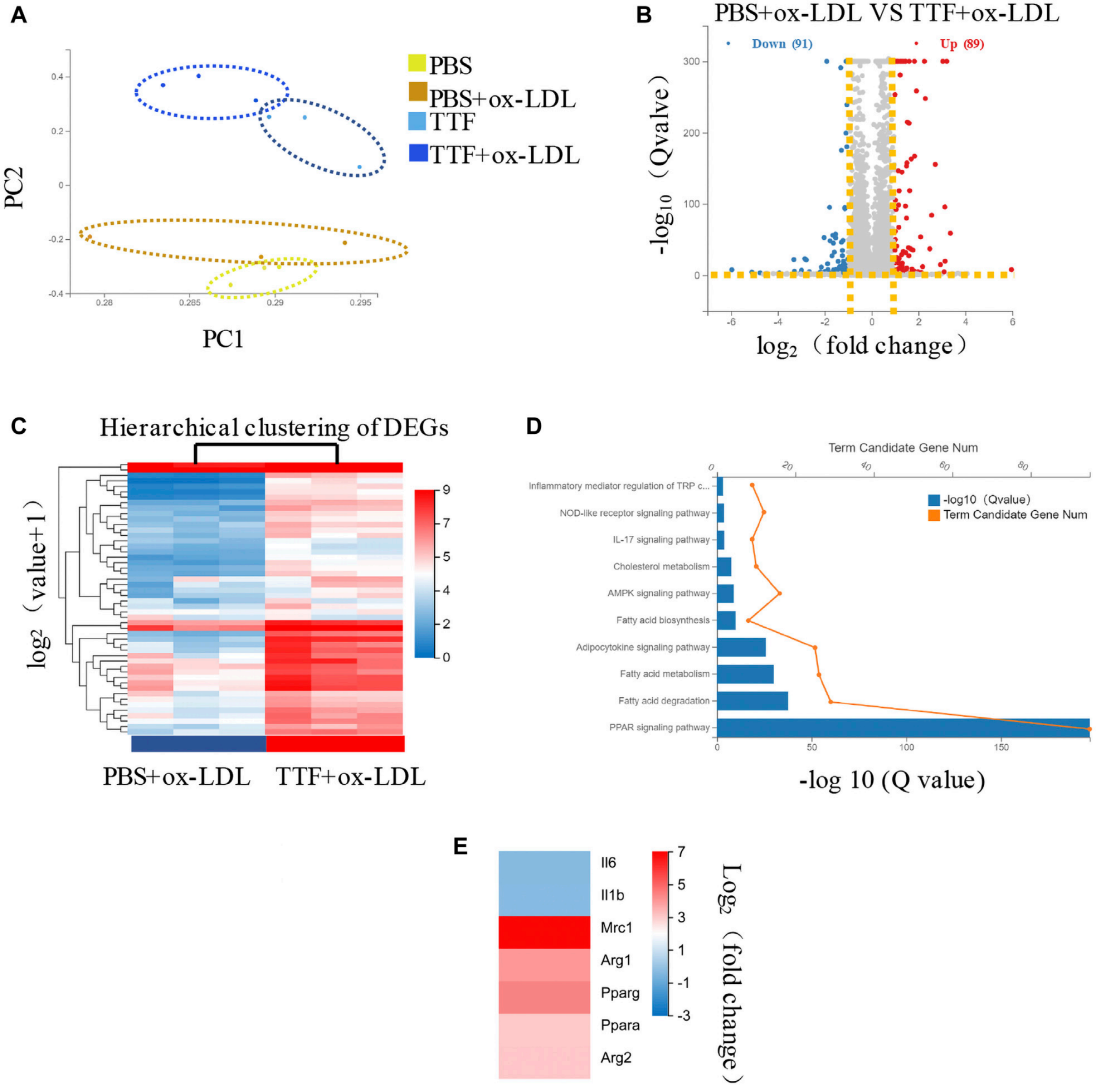
RNA-seq of PM in ApoE^-/-^ mice **(A)** Principal component analysis showed the overall transcriptomic similarity of the four groups **(B)** Differential expression analysis revealed induced by ox-LDL, TTF treatment in macrophages. Volcano plots show the differentially expressed genes (DEGs; fold change >2 or < −2, false discovery rate adjusted *p* value < 0.05) in macrophages (DEGs; fold change >2 or < −2, false discovery rate adjusted *p* value < 0.05) in macrophages **(C)** Hierarchical clustering of quantitative genes expression profiling for macrophages of PBS + ox-LDL and TTF + ox-LDL groups **(D)** KEGG pathway analysis of DEGs between PBS + ox-LDL and TTF + ox-LDL groups **(E)** Selected genes involved in macrophage polarization between TTF + ox-LDL and PBS + ox-LDL groups are shown as a heatmap.

Based on the results described above, TTF regulates M2 polarization in atherosclerotic plaques, but the underlying mechanism was unclear. To determine the specific effect of TTF on the RNA expression of macrophages and related signaling pathways in ApoE^-/-^ mice, we collected PMs from ApoE^-/-^ mice pretreated with TTF and stimulated with ox-LDL. RNA was isolated and subjected to RNA sequencing. Twelve samples were tested, with three samples from each group. Principal component analysis revealed overall transcriptomic similarity among the four groups (Figure 7A). Based on the gene expression levels of each sample, we detected differentially expressed genes between PBS- and TTF-pretreated macrophages induced by ox-LDL using a volcano plot (Figure 7B). The hierarchical clustering of differentially expressed genes are shown in Figure 7C and the results of pathway classification are shown in Figure 7D. Vital genes involved in alternative macrophage activation between TTF + ox-LDL group and PBS + ox-LDL group are shown as a heatmap (Figure 7E). Our results indicate that PPARγ, AMPK, and inflammatory mediator-related signaling pathways may play critical roles in the effects of TTF on the RNA expression of macrophages in ApoE^-/-^ mice.

### TTF Promotes Alternative Macrophage Activation Through PPARγ, AKT/ERK Pathway

**FIGURE 8:**
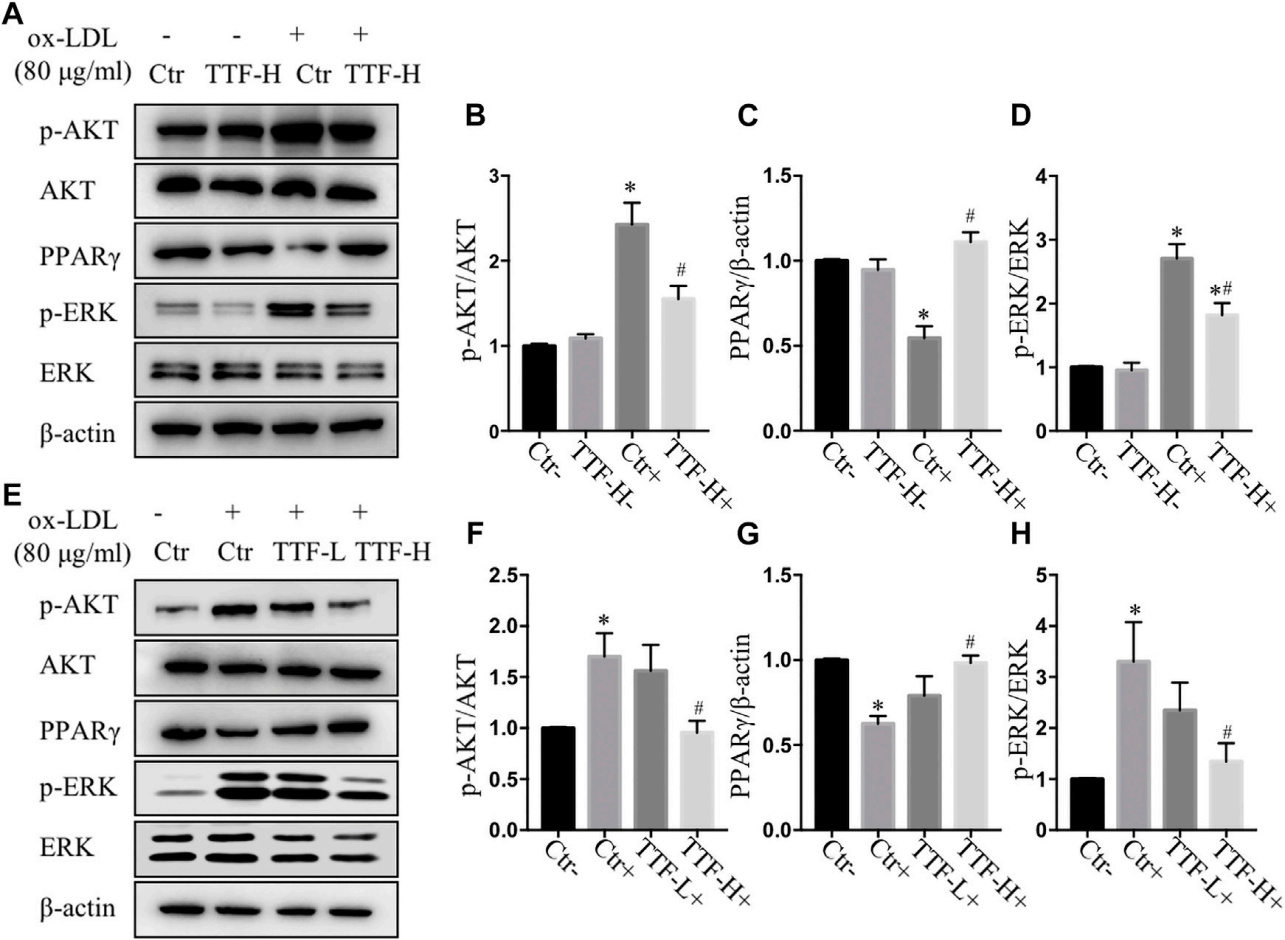
TTF affected atherosclerotic plaque through AKT, ERK and PPARγ signal **(A)** Western blotting showed the protein expression of p-AKT, AKT, PPARγ, p-ERK, and ERK in PM cells treated with PBS and TTF (0.5 mg/ml) stimulated with ox-LDL (80 μg/ml) **(B–D)** The quantitative analysis of grey ratio of p-AKT/AKT, PPARγ/β-actin, and p-ERK/ERK **(E)** Western blotting showed the protein expression of p-AKT, AKT, PPARγ, p-ERK, and ERK in PM cells treated with PBS and TTF (0.25, 0.5 mg/ml) stimulated with ox-LDL (80 μg/ml) **(F–H)** The quantitative analysis of grey ratio of p-AKT/AKT, PPARγ/β-actin, and p-ERK/ERK. Values are presented as means ± SEM (n = 3 per group). *p < 0.05 vs Ctr-group, #*p* < 0.05 vs Ctr + group.

**FIGURE 9:**
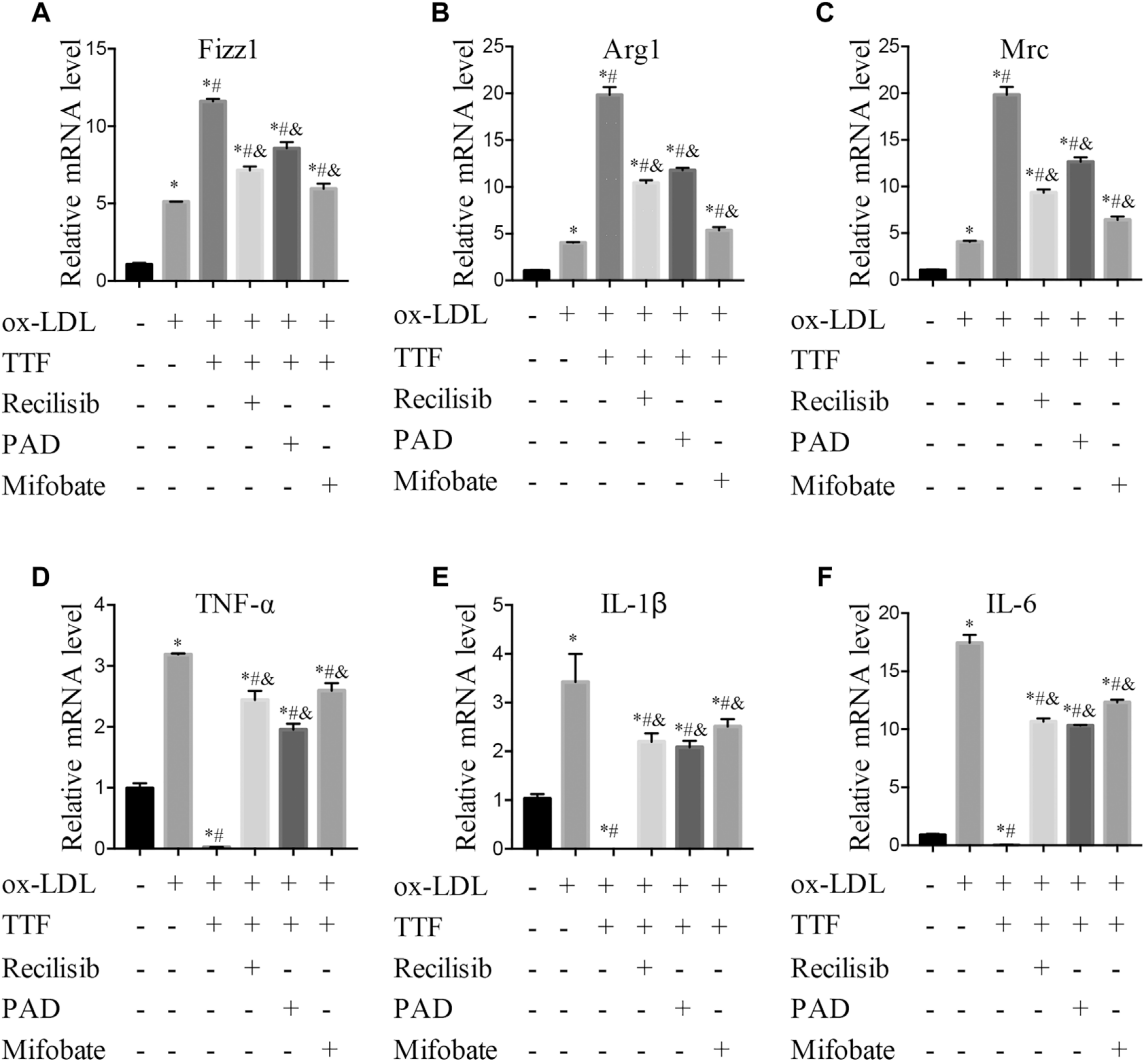
TTF functions through promoting PPARγ signal expression and inhibiting AKT/ERK pathway. PM cells were pretreated with 30 μM AKT activator (Recilisib), 25 nM ERK activator (PAD), or 50 μM PPARγ inhibitor (Mifobate) for 1 h, then treated with 0.5 mg/ml TTF and 80 μg/ml ox-LDL for 24 h. Total RNA was extracted and then relative mRNA level of Fizz1 **(A)**, Arg1 **(B)**, Mrc **(C)**, TNF-α **(D)**, IL-1β **(E)**, and IL-6 **(F)** were detected by q-PCR. Values are presented as means ± SEM (n = 3 per group). *p < 0.05 vs group with ox-LDL only; #*p* < 0.05 vs group without ox-LDL and TTF; &*p* < 0.05 vs group with ox-LDL and TTF.

According to the results of RNA sequencing, to detect the protein expression of key molecules in related signal pathways of TTF affecting macrophage polarization, we performed immunoblotting to determine the protein expression and phosphorylation levels of PPARγ, ERK1/2, and AKT in PMs. We found that 0.5 mg/ml TTF did not affect the expression of the tested proteins in PMs without ox-LDL stimulation. However, upon stimulation with 80 μg/ml ox-LDL, TTF decreased the phosphorylation levels of AKT and ERK and increased the expression of PPARγ significantly (Figures 8A–D). The regulation effect on PPARγ and AKT/ERK was related to the concentration of TTF, as 0.5 mg/ml TTF (TTF-H) had a stronger regulatory effect than 0.25 mg/ml TTF (TTF-L) (Figures 8E–H). This effect was remarkably reversed by an AKT activator (recilisib), ERK activator (pamoic acid disodium, PAD), or PPARγ inhibitor (mifobate). The quantitative PCR results revealed decreased relative mRNA levels of Fizz1, Arg1, and Mrc and increased relative mRNA levels of TNF-α, IL-1β, and IL-6 in PMs pretreated with TTF and 30 μM AKT activator (recilisib), 25 nM ERK activator (PAD), or 50 μM PPARγ inhibitor (mifobate) following stimulation with ox-LDL (Figure 9).

## Discussion

Currently, in terms of modern medicine for the prevention and treatment of stable coronary heart disease, the residual risk of secondary prevention with drugs is high. In addition, the stent implantation is one of the mainstream treatments to cure coronary heart disease. However, after stent implantation, it may still not solve the patient’s main complaint and have symptoms such as angina pectoris remained. Therefore, more economical and effective methods to prevent and treat AS and CHD are urgently needed. In our previous studies, TTF not only delayed plaque progression in an animal AS model, but also enhanced plasma PPARγ in patients with CHD. PPARγ is known to be one of the central links of alternative macrophage activation (Olefsky and Glass, 2010), and macrophage polarization is a critical process in atherosclerosis development and regression (Barrett, 2020; Bonacina et al., 2020). We found that TTF stabilized AS plaques by inducing alternative macrophage activation via the PPARγ and AKT/ERK pathways.

There is a definite interaction between AKT signaling and alternative macrophage activation, and AKT signaling participates in alternative macrophage-enhanced differentiation of periodontal ligament stem cell cementoblastic differentiation (Li et al., 2019). However, alternative macrophage activation functions by activating the phosphorylation of Akt in multiple sclerosis animal models (Weng et al., 2019). The phagocytic ability of alternative macrophages is improved via the Akt/FoxO1 pathway (Liu et al., 2019). In the tumor microenvironment, ERK signaling is positively correlated with alternative macrophage activation (Mu et al., 2018; Cai J. et al., 2020). Insulin modulates macrophage transition from the classic to the alternative macrophage phenotype by upregulating PPARγ expression and inducing P38-mediated dephosphorylation of PPAR-γ (Ser112) (Yu et al., 2019). PPARγ plays an important role in many metabolic diseases but its function remains controversial (Chawla, 2010). It is highly expressed in adipose tissue and atherosclerotic plaques, where it not only promotes the differentiation and storage of lipids, improves insulin sensitivity, and plays an anti-inflammatory role, but also improves the release of nitric oxide synthase from endothelial cells in atherosclerotic plaques and regulates vascular homeostasis (Mirza et al., 2019). However, PPARγ can mediate the expression of pro-inflammatory transcription factors, which can promote AS. In ruptured carotid plaques, macrophage nuclear receptor corepressors can delay plaque progression by inhibiting PPARγ to enhance CD36 scavenger receptor expression (Oppi et al., 2020). Among TCM, numerous botanical drugs can be used to treat AS. Ginsenoside Rg3, one of the main active components of *Panax ginseng*, has been reported to reverse the classic to the alternative macrophage phenotype through PPARγ signaling in diabetic AS mouse models (Guo et al., 2018). In addition to phagocytosis and polarization of macrophages, there are many regulatory necrosis patterns of macrophages in advanced plaques, which are involved in plaque stability (Martinet et al., 2019). (Tao et al., 2020). Therefore, targeting macrophages is an important strategy for treating atherosclerosis.

We established a production process and quality control standard system for TTF granules and liquid extract in line with Good Manufacturing Practice standards. Moreover, based on previous clinical observation studies and animal comparative medicine studies, we conducted A randomized, controlled, multicenter clinical study on the efficacy and safety of tanyutong capsule in the treatment of stable coronary heart disease (phlegm and blood stasis syndrome) (Key R and D project of Zhejiang Provincial Department of science and technology. grant number: 2020C03119) to verify the efficacy and safety of TTF in the prevention and treatment of CHD. In further studies, we will evaluate the specific mechanism of TTF, which would be helpful for the clinical transformation and application of phlegm and blood stasis theory in TCM, and can provide more medication choices for clinical patients with AS Chen et al., 2007.

## Data Availability

The datasets presented in this study can be found in online repositories. The names of the repository/repositories and accession number(s) can be found below: https://www.ncbi.nlm.nih.gov/, SRP326359.

## Abbreviations

TCM, Traditional Chinese Medicine; TTF, Tanyu Tongzhi Formula; HFD, high-fat diet; CHOL, cholesterol; TG, triglyceride; TNF-α, tumor necrosis factor-α; CHD, coronary heart disease; AS, atherosclerosis; PPARγ, peroxisome proliferator-activated receptor γ; GMP, Good Manufacturing Practice.
