# Supplementary material for: Tanyu Tongzhi Formula Delays Atherosclerotic Plaque Progression by Promoting Alternative Macrophage Activation via PPARγ and AKT/ERK Signal Pathway in ApoE Knock-Out Mice
Source: Front Pharmacol. 2021 Dec 13;12:734589. doi: 10.3389/fphar.2021.734589 (PMC8711052; doi:10.3389/fphar.2021.734589)
Supplement: Supplementary file 1 [file Table1.docx]

**Supplementary Table 1 |** Formulation of high-fat diet.

| Class description | Ingredients | grams |
| --- | --- | --- |
| Protein | Casein, Lactic, 30 Mesh | 200.00 g |
| Protein | Cystine, L | 3.00 g |
| Carbohydrate | Starch, Corn | 212.00 g |
| Carbohydrate | Sucrose, Fine Granulated | 124.41 g |
| Carbohydrate | Lodex 10 | 71.00 g |
| Fiber | Solka Floc, FCC200 | 50.00 g |
| Fat | Cocoa Butter, Deodorized | 155.00 g |
| Fat | Soybean Oil, USP | 25.00 g |
| Mineral | Potassium Citrate, Monohydrate | 16.50 g |
| Mineral | Calcium Phosphate, Dibasic | 13.00 g |
| Mineral | Calcium Carbonate, Light, USP | 5.50 g |
| Mineral | S10020 | 5.00 g |
| Mineral | Sodium Chloride | 2.59 g |
| Vitamin | Choline Bitartrate | 2.00 g |
| Vitamin | V10001C | 1.00 g |
| Special | Cholesterol, NF | 11.25 g |
| Dye | Dye, Blue FD&C #1, Alum. Lake 35-42% | 0.05 g |
| Dye | Dye, Yellow FD&C #5, Alum. Lake 35-42% | 0.05 g |
|  | Total: | 897.35 g |
